# Antifungal Activity of Bulgarian Rose Damascena Oil against Vaginitis-Causing Opportunistic Fungi

**DOI:** 10.1155/2023/5054865

**Published:** 2023-12-01

**Authors:** Yejin Lee, Eunhye Park, Bohee Jang, Jisun Hwang, Jinmin Lee, Eok-Soo Oh

**Affiliations:** ^1^Department of Life Sciences, Ewha Womans University, Seoul 03760, Republic of Korea; ^2^Jayeonin Inc., Seoul 04995, Republic of Korea

## Abstract

Since Bulgarian rose damascena oil is known for its anti-inflammatory, antioxidant, and antimicrobial properties, we investigated its antifungal activity against the species of *Candida*, which are among the most common opportunistic fungal pathogens. Our disk-diffusion assay revealed that Bulgarian rose damascena oil effectively inhibited the growth of *Candida albicans* along with various bacteria. The minimum inhibitory and fungicidal concentrations against *Candida albicans* and *Candida glabrata* were all 0.25%. Under our experimental conditions, Bulgarian rose damascena oil showed better inhibitory effects on *Candida glabrata* and *Candida albicans* than several popular essential oils reported to have antifungal activity other than *Origanum vulgare* oil. Interestingly, Bulgarian rose damascena oil showed better antifungal activity against *Candida* species at acidic pH and induced cell death of *Candida* species in the culture medium, with cell death seen in 25–35% of the cells exposed to 0.05% Bulgarian rose damascena oil. Furthermore, Bulgarian rose damascena oil inhibited the hyphal growth of *Candida albicans* cultured in the RPMI medium with fetal bovine serum. These findings collectively suggest that Bulgarian rose damascena oil has antifungal activity against *Candida* species and thus could potentially be developed in novel therapies for vaginitis-causing pathogenic fungi.

## 1. Introduction

Pathogenic fungi cause various diseases in humans and other organisms [1]. Some of them cause relatively mild skin infections, while others cause serious skin infections or life-threatening systemic infections [2]. One of the most common systemic infections worldwide is caused by *Candida* species [3]. Human *Candida* species inhabit various body surfaces (e.g., the oral cavity, gastrointestinal tract, vagina, and skin) of healthy persons as a commensal organism [4] and cause infections or candidiasis in superficial mucous membranes [3]. A common female disease is vaginitis, which is an inflammation of the vagina caused by *Candida* species [5]. Vulvovaginal candidiasis (VVC), which is a vaginal yeast infection caused by *Candida* species, is the leading cause of vaginitis [6]. Of the various *Candida* species, only a few are opportunistic pathogens in humans [3]. *Candida albicans* is the most common pathogen causing VVC, and other non-*albicans Candida* appear to be on the increase [7, 8]. Of the non*-albicans Candida*, *Candida glabrata*, which was considered a relatively nonpathogenic saprophyte in the normal flora of healthy individuals [9], is regarded as the second most common cause of VVC [10]. When the balance between the vaginal colonization of *Candida* and the host environment is disturbed, a weak immune system can allow pathogens to cause VVC [3, 7]. The most widely available treatments are the local applications of nystatin or topical azoles (e.g., clotrimazole, butoconazole, and miconazole) or triazoles (e.g., fluconazole) [6, 11, 12]. Both oral fluconazole and vaginal nystatin are considered to be particularly effective in treating VVC [6]. However, the increased use of that immunosuppressive therapy together with broad-spectrum antimycotic therapy leads to an increased frequency of mucosal and systemic infections caused by *Candida* species, particularly *Candida glabrata* [13].

The paucity of effective antifungal agents and the emergence of drug resistance have prompted researchers to seek novel antifungal agents [12, 14, 15]. Extracts of various plants, including herbs and traditional medicinal plants, have long been known to offer antifungal activities. For instance, *Acacia nilotica* and *Coriandrum sativum* have been found to possess antifungal activities that decrease biofilm growth or inhibit the synthesis of cell wall components by yeast [16, 17]. Importantly, various essential oils, including *Origanum vulgare*, *Ocimum basilicum*, *Lavandula*, *Mentha spicata*, *Mentha piperita*, and *Melaleuca alternifolia* essential oils, have been shown to inhibit the fungal growth and activity of *Candida albicans* more efficiently than the known antifungal drugs, including clotrimazole [18].

*Rosa damascena* is a small plant with aromatic pale pink flowers, also known as Bulgarian rose or Damask rose; it is mainly applied for its perfuming effects [19, 20]. However, *Rosa damascena* also contains flavonoids, such as kaempferol, quercetin, terpenes, tannins, and vitamin C [19–21], and its essential oil has been used as a natural medicine to treat inflammation, coughs [19, 22], and digestive problems [23]. Recently, Bulgarian rose damascena oil was reported to confer broad-spectrum antimicrobial activities, including antibacterial effects against *Staphylococcus aureus*, *Bacillus subtilis*, *Pseudomonas aeruginosa*, and *Escherichia coli* [19, 21], as well as antiviral activity [24]. In the present study, we investigated the antifungal effects of Bulgarian rose damascena oil against *Candida albicans* and *Candida glabrata*.

## 2. Materials and Methods

### 2.1. Strains and Media

Luria broth (LB) and potato dextrose agar (PDA) were purchased from Alpha Biosciences (Baltimore, MD, USA). *Staphylococcus aureus* KCTC1621, *Staphylococcus epidermidis* KCTC1917, and *Pseudomonas aeruginosa* KCTC1636 were purchased from Korean Collection for Type Cultures (KCTC, Jeongeup, Korea). *Bacillus subtilis* KACC14741 and *Candida albicans* strains KACC 30003 and KACC 30004 were purchased from Korean Agricultural Culture Collection (KACC, Joenju, Korea). *Escherichia coli* DH5*α* was purchased from RBC Bioscience (Taiwan). *Candida glabrata* strains KBNO6P00368 and KBNO6P00369 were obtained from Chonbuk National University Hospital (Cheongju, Korea). The Gram-positive (*Staphylococcus aureus*, *Bacillus subtilis*, and *Staphylococcus epidermidis*) and Gram-negative (*Escherichia coli* and *Pseudomonas aeruginosa*) bacterial strains were cultured at 37°C under constant shaking (200 rpm) in the complete liquid medium (LB) consisting of 10 g/L enzymatic digest of casein, 5 g/L yeast extract, and 10 g/L sodium chloride. Fungi (*Candida glabrata* and *Candida albicans*) were cultured at 37°C under constant shaking (200 rpm) in the complete liquid medium (PDA) consisting of 4 g/L potato infusion and 20 g/L dextrose.

### 2.2. Materials

The essential oils made by steam distillation of *Origanum vulgare*, *Ocimum basilicum*, *Mentha spicata*, and *Eucalyptus* were purchased from Botanic Story (Brea, CA, USA) and those of *Melaleuca alternifolia, Mentha piperita*, and *Lavandula* were purchased from doTERRA (Pleasant Grove, UT, USA). Bulgarian rose damascena oil was purchased from Enio Bonchev Production (Sofia, Bulgaria). Trypan blue stain (0.4%) was purchased from Gibco (Waltham, MA, USA). Ampicillin and fluconazole were purchased from Sigma-Aldrich (St. Louis, MO, USA).

### 2.3. Determination of the Antibacterial Activity by the Disk-Diffusion Method

The antibacterial and antifungal activities of the Bulgarian rose damascena oil against microorganisms were examined by the standard method of the European Committee on Antibiotic Susceptibility Testing (EUCAST) disk-diffusion assay [25, 26]. After determining the bacterial count with spectrophotometric (turbidimetric) analysis by measuring absorbance at 600 nm with the microplate reader (Molecular devices, San Jose, CA, USA), microorganisms with a total number of 1 × 10^6^ in 40 *μ*L were spread onto LB or PDA plates and sterile 8-mm paper disks (Advantec, Tokyo, Japan) were placed onto the plates. All essential oils were dissolved in 70% ethanol at a concentration varying from 0 to 10% (v/v), and 40 *μ*L of each sample was applied to discs and then the plates were incubated at 37°C for 24 h. The inhibition zones were measured in millimeters using a Vernier caliper. Ampicillin (50 mg/ml), fluconazole (1 mg/ml and 2 mg/ml), and 70% ethanol were used as positive controls for bacteria and yeasts, respectively.

### 2.4. Determination of the Minimum Inhibitory Concentrations (MICs) by the Microbroth-Dilution Method

Serial doubling dilutions of Bulgarian rose damascena oil with the PDA liquid medium at concentrations ranging from 0 to 0.25% were prepared. The wells of a 96-well plate were filled with 50 *μ*L/well of each Bulgarian rose damascena oil dilution and 50 *μ*L/well of each *Candida* species with a total number of 1 × 10^3^ cells/*μ*L. The plates were incubated for 24 h at 37°C, and the MIC was determined by measuring absorbance at 600 nm. The MIC endpoint was taken as the lowest concentration of Bulgarian rose damascena oil with which no growth was detected, as determined by the EUCAST disk-diffusion assay [25, 26].

### 2.5. Determination of the Minimum Fungicidal Concentrations (MFCs)

After the determination of the MIC of the Bulgarian rose damascena oil, media from each well that lacked visible fungal growth were replicated on PDA plates and incubated for 24 h at 37°C. The lowest concentration at which 99.9% of the fungal population was killed in PDA plates was taken as the MFC endpoint [26].

### 2.6. Influence of pH on the Antifungal Activity of Bulgarian Rose Damascena Oil

*Candida* species were incubated in the PDA liquid medium (pH 5.4) and 10 *µ*L of each culture (1 × 10^3^ cells/*μ*L) was mixed with 90 *µ*L of the PDA liquid medium with different pH values (pH 5.1, 5.4, 5.7, and 6.0), resulting in a final concentration below the MIC (0.125%) of Bulgarian rose damascena oil. After 24 h incubation, the growth of *Candida* species was determined based on the optical density of the samples.

### 2.7. Trypan Blue Staining

Media containing *Candida* were diluted with the same volume of 0.4% trypan blue solution and incubated for 3 min at room temperature. Each sample was loaded to a hemocytometer, the cells were counted under a microscope, and the average number of stained cells was determined [27].

### 2.8. Hyphal Growth Test

*C. albicans* species (1 × 10^5^ cell/mL) were incubated at 37°C in RPMI 1640 for 24 h at 37°C in the absence or presence of different concentrations of Bulgarian rose damascena oil or 10% (v/v) fetal bovine serum (FBS). Each sample was mixed with an equal volume of trypan blue, and 10 *μ*L of the mixture was transferred to the hemocytometer and viewed under light microscopy to assess hyphal formation.

### 2.9. Statistical Analysis

Data are presented as the mean ± SD (standard deviation) for each independent experiment. Statistical analyses were performed using an unpaired Student's *t*-test. A value of *p* < 0.05 was considered to represent a statistically significant difference.

## 3. Results

### 3.1. Antibacterial and Antifungal Activities of Bulgarian Rose Damascena Oil

**Figure 1:**
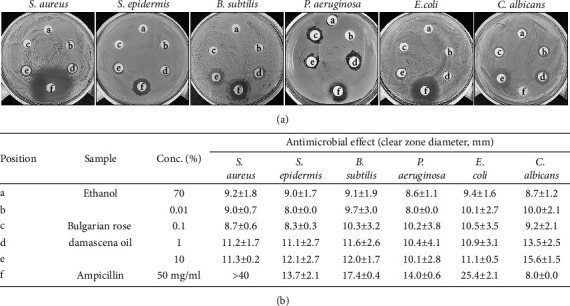
Antimicrobial activity of Bulgarian rose damascena oil. The disk-diffusion assay of Bulgarian rose damascena oil against *Staphylococcus aureus*, *Staphylococcus epidermidis*, *Bacillus subtilis*, *Pseudomonas aeruginosa*, *Escherichia coli*, and *Candida albicans.*

To investigate the antibacterial and antifungal activities of Bulgarian rose damascena oil, we performed disk-diffusion assays with five strains of bacteria and one strain of fungus (Figure 1). Bulgarian rose damascena oil dissolved in 70% ethanol was applied to the bacteria strains and the formation of a clear zone was examined after 24 h; ampicillin (final 2 mg/ml) and 70% ethanol were used as positive controls for Gram-positive and Gram-negative bacteria (Figure 1(a)). Our results revealed that 1% Bulgarian rose damascena oil effectively inhibited the growth of *Staphylococcus aureus, Staphylococcus epidermidis, Bacillus subtilis*, *Pseudomonas aeruginosa*, and *Escherichia coli* with inhibition zones of 10–12 mm diameter, confirming the antibacterial properties of the Bulgarian rose damascena oil. We further found that 1% Bulgarian rose damascena oil effectively inhibited *Candida albicans* with an inhibition zone of 13.5 mm, whereas 50 mg/ml ampicillin, an antibacterial antibiotic, did not inhibit the growth of *Candida albicans* (Figure 1(b)).

**Figure 2:**
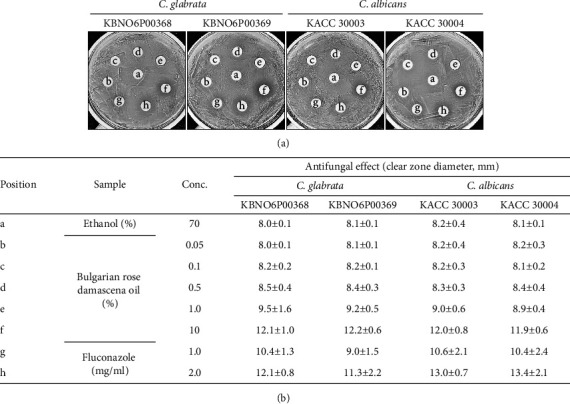
Antifungal activity of Bulgarian rose damascena oil against *Candida* species. The disk-diffusion analysis of Bulgarian rose damascena oil against *Candida* species.

To examine the antifungal properties of Bulgarian rose damascena oil against *Candida albicans* and *Candida glabrata*, which are human opportunistic pathogens of VVC [3], a disk-diffusion test was performed with two strains of *Candida glabrata* and two strains of *Candida albicans* (Figure 2). In the case of *Candida glabrata* KBNO6P00368, Bulgarian rose damascena oil induced the formation of clear zones with diameters of 8.2 ± 0.2 mm at 0.1%, 8.5 ± 0.4 mm at 0.5%, 9.5 ± 1.6 mm at 1.0%, and 12.1 ± 1.0 mm at 10%, suggesting that Bulgarian rose damascena oil inhibited the growth of *Candida glabrata* KBNO6P00368 in a dose-dependent manner (Figure 2(b)). Similar results were obtained with *Candida glabrata* KBNO6P00369 and *Candida albicans* KACC 30003 and KACC 30004 (Figure 2(b)). Interestingly, the antifungal activity of 10% Bulgarian rose damascena oil against all the test strains was greater than the activity of 2 mg/ml *fluconazole,* a known antifungal drug (Figure 2(b)).

### 3.2. Determination of the Minimum Inhibitory Concentration and Minimum Fungicidal Concentration for Bulgarian Rose Damascena Oil against *Candida* Species

**Figure 3:**
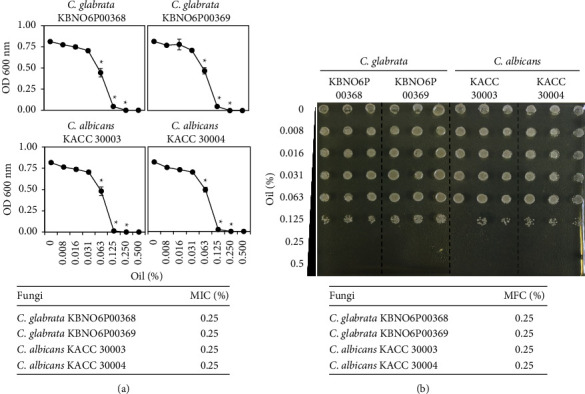
The MIC and MFC of Bulgarian rose damascena oil against *Candida* species. (a) *Candida* species were cultured in the PDA liquid medium containing the indicated concentrations of Bulgarian rose damascena oil for 24 h and the absorbance of microtiter plates was measured at 600 nm (top panel). (b) MFC determination with replica plate of all the wells lacking fungal growth. The MFC was determined as the lowest concentration of Bulgarian rose damascena oil at which no visible colony formation was detected (bottom panel).

To further assess the antifungal potential of Bulgarian rose damascena oil, the microdilution method was used to determine the minimum inhibitory concentrations (MICs) and the minimum fungicidal concentrations (MFCs) values (Figure 3). The MIC value for Bulgarian rose damascena oil was 0.25% against both *Candida glabrata* and *Candida* albicans (Figure 3(a)). Thereafter, media from all Bulgarian rose damascena oil-treated wells showing no visible fungal growth were replicated on PDA plates for the determination of the MFC. The calculated MFC was 0.25% for both *Candida glabrata* and *Candida* albicans (Figure 3(b)).

### 3.3. pH Dependence of the Antifungal Activity of *Rosa damascena* Essential Oil

**Figure 4:**
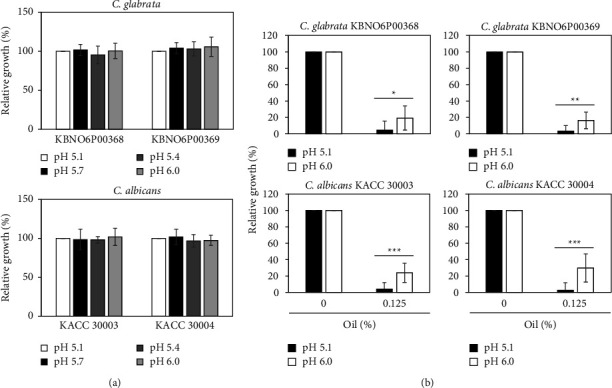
pH dependence of the antifungal effect of Bulgarian rose damascena oil. In the absence (a) or presence (b) of the indicated concentrations of Bulgarian rose damascena oil, for 24 h, the growth of *Candida* species was determined by measuring the absorbance at 600 nm (^*∗*^*p* < 0.05, ^*∗∗*^*p* < 0.01, and ^*∗∗∗*^*p* < 0.001).

**Figure 5:**
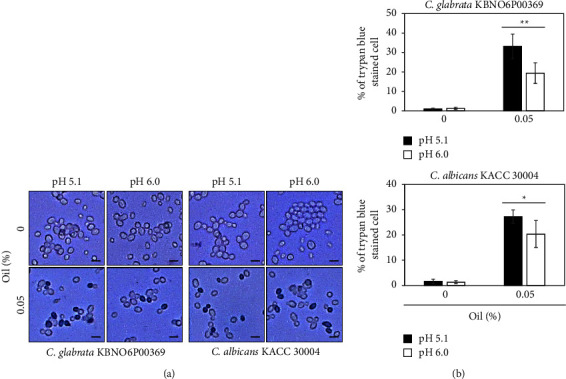
Effect of Bulgarian rose damascena oil on the cell death of *Candida* species at different pH levels. *Candida* species were incubated in the PDA medium at different pH values with 0.05% of Bulgarian rose damascene oil for 24 h and stained. Cells were examined under a microscope. Scale bars = 5 *µ*m (a) and counted (b).

Since the antibacterial properties of natural antibacterial materials are known to depend on pH [28, 29], we next investigated the antifungal activity of Bulgarian rose damascena oil in media of different pH values (Figure 4). Our data revealed that fungal growth was very similar in a range of pH 5.1–6.0 among the four strains of *Candida* species (Figure 4(a)), but the antifungal activity of the Bulgarian rose damascena oil differed across this range of pH. When applied at a concentration of 0.125%, the Bulgarian rose damascena oil more effectively inhibited the growth of all *Candida* species cultured on the PDA media of pH 5.1 versus the PDA media of pH 6.0 (Figure 4(b)). To further confirm this finding, *Candida* species treated with Bulgarian rose damascena oil were stained with trypan blue and the dead (stained) cells were counted (Figure 5). Since 0.125% of Bulgarian rose damascena oil effectively inhibited the growth of all *Candida* species, to quantify cell death, we lowered the amount of Bulgarian rose damascena oil into 0.05%. In the PDA medium of pH 5.1 lacking the essential oil, we did not observe any trypan blue staining of *Candida* species. In contrast, around 25–35% of *Candida glabrata* and *Candida* albicans cells treated with 0.05% Bulgarian rose damascena oil exhibited trypan blue staining (Figure 5), supporting the idea that Bulgarian rose damascena oil substantially induces cell death of *Candida* species (Figure 5(b)). This effect was greatly reduced against *Candida* species grown in the PDA medium of pH 6.0 (Figure 5(b)), further supporting our contention that the antifungal activity of Bulgarian rose damascena oil is pH dependent.

### 3.4. Inhibitory Effect of Bulgarian Rose Damascena Oil on the Hyphal Growth of *Candida albicans*

**Figure 6:**
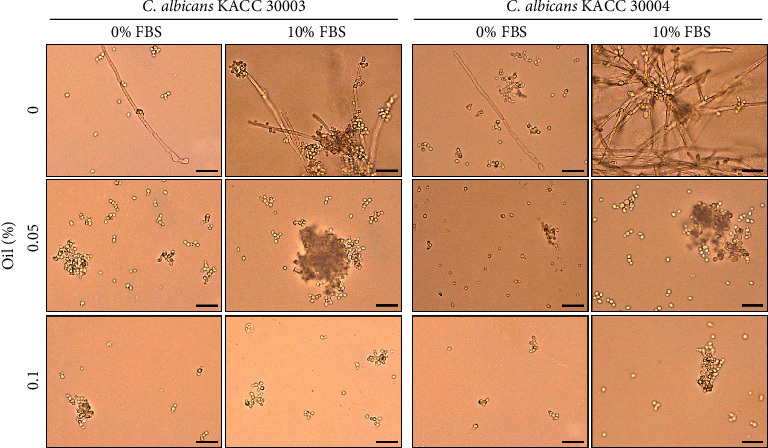
Effects of Bulgarian rose damascena oil on hyphal development. *Candida albicans* was incubated in the RPMI medium containing 0% or 10% FBS in the presence of the indicated concentrations of Bulgarian rose damascena oil for 24 h. Bright-field microscopic images are shown. Scale bars = 20 *μ*m.

**Figure 7:**
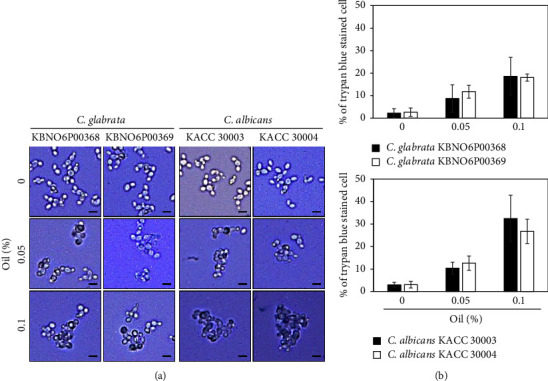
Cell death of *Candida* species treated with Bulgarian rose damascena oil. *Candida* species were incubated in the RPMI medium at 0.05% and 0.1% of Bulgarian rose damascene oil for 24 h and stained. Cells were examined under a microscope (scale bars = 5 *μ*m (a) and counted (b)).

*Candida albicans* can penetrate the human epithelial and endothelial cells during the early stages of infection and cause damage through the formation of certain morphological forms, such as hyphae [30–33]. Thus, we next investigated the ability of Bulgarian rose damascena oil to inhibit the formation of hyphae (Figure 6). *Candida albicans* cells were cultured in the RPMI medium and the formation of hyphae was examined. A small number of hyphae was formed in *Candida albicans* cultured in RPMI without FBS, whereas elongated hyphal forms were seen among most of the *Candida albicans* cells cultured in RPMI containing 10% FBS, which is a commonly used hyphal inductor. Bulgarian rose damascena oil (at both 0.05% and 0.1%) completely abolished the hyphal formation induced by 10% FBS (Figure 6). When *Candida* species cultured in the RPMI medium were stained with trypan blue (Figure 7), <5% of *Candida glabrata* or *Candida albicans* cells were stained in the absence of Bulgarian rose damascena oil, and this proportion was concentration-dependently increased by treatment with Bulgarian rose damascena oil, suggesting that treatment with Bulgarian rose damascena oil could decrease the infectivity of this pathogen by decreasing hyphal formation and killing cells.

### 3.5. Comparative Analysis of the Antifungal Activity of Various Essential Oils against *Candida* Species

Essential oils extracted from various plants are well-known as antimicrobial agents that can exhibit a broad spectrum of activities, including antifungal properties [17, 18], with antifungal MIC values of 0.25∼4% (v/v) [34–36]. We, therefore, further compared the efficacies of Bulgarian rose damascena oil and other essential oils (*Origanum vulgare*, *Ocimum basilicum*, *Mentha spicata*, *Melaleuca alternifolia*, *Mentha piperita*, *Eucalyptus*, and *Lavandula*) against *Candida* species [34–36] by the disk-diffusion assay. Under our experimental conditions with 2% in the midrange to compare different essential oils, the ability of Bulgarian rose damascena oil to inhibit the tested strains of *Candida glabrata* and *Candida albicans* was comparable to those of *Mentha piperita* oil, whereas the other tested oils except *Origanum vulgare* oil showed weaker inhibitory effects (Table 1).

## 4. Discussion

Although a variety of natural products, including essential oils, have been found to possess antifungal activity [34–36], no previous study had examined the potential action of Bulgarian rose damascena oil against *Candida albicans* and *Candida glabrata*, which are human opportunistic pathogens that can cause VVC [3]. In this study, we demonstrate that Bulgarian rose damascena oil had antifungal activity against *Candida* species, including *Candida glabrata* and *Candida albicans*, with the MIC and MFC of 0.25% (Figure 3). Consistently, 0.05% Bulgarian rose damascena oil induced cell death of 25–35% of *Candida* cells of two different species (Figure 5).

As natural antimicrobial agents, essential oils exert a broad spectrum of activities, including antifungal properties [18, 34, 35]. *Origanum vulgare*, *Ocimum basilicum*, *Mentha spicata*, *Melaleuca alternifolia*, *Mentha piperita*, *Eucalyptus*, and *Lavandula* essential oils have been shown to be particularly effective against fungi, with most of them exhibiting the MIC of 0.25∼4% (v/v) [34–36]. Among the essential oils tested herein, Bulgarian rose damascena oil showed a strong inhibitory effect on all of *Candida glabrata* and *Candida albicans* that was better than those of all other tested essential oils except for *Origanum vulgare* oil, which showed a comparable activity (Table 1). Consistent with this, Bulgarian rose damascena oil induced cell death of *Candida* species in culture media (Figure 5) and inhibited the hyphal growth of *Candida albicans* cultured in RPMI media (Figure 7). Based on these findings, we suggest that Bulgarian rose damascena oil could be developed for novel therapies against human pathogenic fungi that may be applied in cosmetic formulations. For instance, ginger and lemon essential oils are key ingredients of many antiacne creams because of their antibacterial activity against *Propionibacterium acnes*, and lemon essential oils have an antioxidant activity that could have antiaging effects [37, 38]. In addition, since essential oils can deter insects, many essential oils are used as mosquito repellents [38, 39].

Natural products, such as Bulgarian rose damascena oil, offer significant advantages for commercial use. For example, vaginitis is characterized by inflammation of the vagina, which can lead to discharge, itching, and pain [3, 7, 11]. One of the most common causes of vulvovaginal itching and discharge is the infection from *Candida* types [6]. At present, various compounds (including antibiotics) are mixed together to efficiently overcome the symptoms of candidiasis [6, 11, 12]. However, the use of Bulgarian rose damascena oil could provide safe, convenient, and effective antifungal effects in this context (Table 1). Especially considering that the normal vaginal pH level is moderately acidic and the antifungal properties of Bulgarian rose damascena oil are stronger against both *Candida glabrata* and *Candida albicans* at acidic pH, Bulgarian rose damascena oil could provide alternatives therapies for vaginal candidiasis. Besides, given that essential oils are the foundation of many cosmetics [37–39], Bulgarian rose damascena oil could also prove very valuable in the cosmetic industry.

In summary, we herein show that Bulgarian rose damascena oil exhibits antifungal activity against *Candida* species that can cause vaginitis. Although further studies will be required to fully elucidate the mechanism underlying Bulgarian rose damascena oil-mediated antifungal activity, our present findings could potentially facilitate research for new alternatives or complementary therapies against vaginal candidiasis.

## 5. Conclusion

We herein show that Bulgarian rose damascena oil exhibits antifungal activity against *Candida* species that can cause vaginitis. Although further studies will be required to fully elucidate the mechanism underlying Bulgarian rose damascena oil-mediated antifungal activity, our present findings could potentially facilitate research for new alternatives or complementary therapies against vaginal candidiasis.

## Figures and Tables

**Table 1:** Efficacy of Bulgarian rose damascena oil against *Candida* species.

Essential oils	Concs. (%)	Antifungal effect (clear zone diameter, mm)			
		*C. glabrata*		*C. albicans*	
		KBNO6P 00368	KBNO6P 00369	KACC 30003	KACC 30004
*Origanum vulgare*	2	17.3 ± 0.4	17.4 ± 0.2	17.4 ± 0.2	17.3 ± 0.4
*Ocimum basilicum*	2	8.7 ± 0.6	8.9 ± 0.6	9.5 ± 0.5	9.6 ± 0.1
*Mentha spicata*	2	8.6 ± 0.6	8.6 ± 0.1	8.5 ± 0.2	8.4 ± 0.1
*Melaleuca alternifolia*	2	8.9 ± 0.8	8.2 ± 0.0	8.9 ± 0.4	9.8 ± 1.1
*Mentha* × *piperita*	2	10.3 ± 0.7	9.2 ± 0.3	9.5 ± 0.7	10.2 ± 0.4
*Eucalyptus*	2	8.9 ± 1.2	8.1 ± 0.1	8.3 ± 0.0	8.4 ± 0.2
*Lavandula*	2	8.9 ± 0.1	9.1 ± 0.4	9.5 ± 0.1	10.2 ± 1.0
Bulgarian rose damascena	2	9.3 ± 0.1	9.8 ± 0.4	10.0 ± 0.6	12.0 ± 0.3
Ethanol	70	8.0 ± 0.0	8.0 ± 0.0	8.0 ± 0.0	8.0 ± 0.0

## Data Availability

The data used to support the findings of this study are available from the corresponding author upon request.

## References

[1] Kohler J. R., Casadevall A., Perfect J. (2014). The spectrum of fungi that infects humans. *Cold Spring Harbor Perspectives in Medicine*.

[2] Pathakumari B., Liang G., Liu W. (2020). Immune defence to invasive fungal infections: a comprehensive review. *Biomedicine and Pharmacotherapy*.

[3] Spampinato C., Leonardi D. (2013). Candida infections, causes, targets, and resistance mechanisms: traditional and alternative antifungal agents. *BioMed Research International*.

[4] Sharma N., Bhatia S., Singh Sodhi A., Batra N. (2018). Oral microbiome and health. *AIMS Microbiology*.

[5] Egan M. E., Lipsky M. S. (2000). Diagnosis of vaginitis. *American Family Physician*.

[6] Dovnik A., Golle A., Novak D., Arko D., Takač I. (2015). Treatment of vulvovaginal candidiasis: a review of the literature. *Acta Dermatovenerologica Alpina Pannonica et Adriatica*.

[7] Cassone A. (2015). Vulvovaginal *Candida albicans* infections: pathogenesis, immunity and vaccine prospects. *BJOG*.

[8] Goncalves B., Ferreira C., Alves C. T., Henriques M., Azeredo J., Silva S. (2016). Vulvovaginal candidiasis: epidemiology, microbiology and risk factors. *Critical Reviews in Microbiology*.

[9] Fidel P. L., Vazquez J. A., Sobel J. D. (1999). Candida glabrata: review of epidemiology, pathogenesis, and clinical disease with comparison to C. albicans. *Clinical Microbiology Reviews*.

[10] Willems H. M. E., Ahmed S. S., Liu J., Xu Z., Peters B. M. (2020). Vulvovaginal candidiasis: a current understanding and burning questions. *Journal of Fungi*.

[11] Sobel J. D. (2007). Vulvovaginal candidosis. *The Lancet*.

[12] Srinivasan A., Lopez-Ribot J. L., Ramasubramanian A. K. (2014). Overcoming antifungal resistance. *Drug Discovery Today: Technologies*.

[13] Pappas P. G., Lionakis M. S., Arendrup M. C., Ostrosky-Zeichner L., Kullberg B. J. (2018). Invasive candidiasis. *Nature Reviews Disease Primers*.

[14] Robbins N., Wright G. D., Cowen L. E. (2016). Antifungal drugs: the current armamentarium and development of new agents. *Microbiology Spectrum*.

[15] Sardi J. C. O., Scorzoni L., Bernardi T., Fusco-Almeida A. M., Mendes Giannini M. J. S. (2013). Candida species: current epidemiology, pathogenicity, biofilm formation, natural antifungal products and new therapeutic options. *Journal of Medical Microbiology*.

[16] Soliman S., Alnajdy D., El-Keblawy A. A., Mosa K. A., Khoder G., Noreddin A. M. (2017). Plants’ natural products as alternative promising anti-Candida drugs. *Pharmacognosy Reviews*.

[17] Zida A., Bamba S., Yacouba A., Ouedraogo-Traore R., Guiguemde R. T. (2017). Anti-Candida albicans natural products, sources of new antifungal drugs: a review. *Journal de Mycologie Médicale*.

[18] Nazzaro F., Fratianni F., Coppola R., Feo V. (2017). Essential oils and antifungal activity. *Pharmaceuticals*.

[19] Boskabady M. H., Shafei M. N., Saberi Z., Amini S. (2011). Pharmacological effects of rosa damascena. *Iranian Journal of Basic Medical Sciences*.

[20] Mileva M., Ilieva Y., Jovtchev G. (2021). Rose flowers-A delicate perfume or a natural healer?. *Biomolecules*.

[21] Labban L., Thallaj N. (2020). The medicinal and pharmacological properties of damascena rose (*Rosa damascena*): a review. *International Journal of Herbal Medicine*.

[22] Mahboubi M. (2016). Rosa damascena as holy ancient herb with novel applications. *Journal of Traditional and Complementary Medicine*.

[23] Akram M., Riaz M., Munir N. (2020). Chemical constituents, experimental and clinical pharmacology of Rosa damascena: a literature review. *Journal of Pharmacy and Pharmacology*.

[24] Vilhelmova-Ilieva N., Dobreva A., Doynovska R., Krastev D., Mileva M. (2021). Antiviral activity of rosa damascena mill. And rosa alba L. Essential oils against the multiplication of herpes simplex virus type 1 strains sensitive and resistant to acyclovir. *Biology*.

[25] Shin J. H. (2009). Current status of antifungal susceptibility testing: methods and clinical application. *Korean Journal of Clinical Microbiology*.

[26] Park B. J., Arthington-Skaggs B. A., Hajjeh R. A. (2006). Evaluation of amphotericin B interpretive breakpoints for Candida bloodstream isolates by correlation with therapeutic outcome. *Antimicrobial Agents and Chemotherapy*.

[27] Smith S. E., Csank C., Reyes G., Ghannoum M. A., Berlin V. (2002). Candida albicans RHO1 is required for cell viability in vitro and in vivo. *FEMS Yeast Research*.

[28] Chaillot J., Tebbji F., García C., Wurtele H., Pelletier R., Sellam A. (2017). pH-dependant antifungal activity of valproic acid against the human fungal pathogen Candida albicans. *Frontiers in Microbiology*.

[29] Valle-Gonzalez E. R., Jackman J. A., Yoon B. K., Mokrzecka N., Cho N. J. (2020). pH-dependent antibacterial activity of glycolic acid: implications for anti-acne formulations. *Scientific Reports*.

[30] Chen H., Zhou X., Ren B., Cheng L. (2020). The regulation of hyphae growth in Candida albicans. *Virulence*.

[31] Naglik J. R., Moyes D. L., Wachtler B., Hube B. (2011). Candida albicans interactions with epithelial cells and mucosal immunity. *Microbes and Infection*.

[32] Sobel J. D., Muller G., Buckley H. R. (1984). Critical role of germ tube formation in the pathogenesis of candidal vaginitis. *Infection and Immunity*.

[33] Sudbery P., Gow N., Berman J. (2004). The distinct morphogenic states of Candida albicans. *Trends in Microbiology*.

[34] Bona E., Cantamessa S., Pavan M. (2016). Sensitivity of Candida albicans to essential oils: are they an alternative to antifungal agents?. *Journal of Applied Microbiology*.

[35] Rajkowska K., Otlewska A., Kunicka-Styczyńska A., Krajewska A. (2017). Candida albicans impairments induced by peppermint and clove oils at sub-inhibitory concentrations. *International Journal of Molecular Sciences*.

[36] Tampieri M. P., Galuppi R., Macchioni F. (2005). The inhibition of Candida albicans by selected essential oils and their major components. *Mycopathologia*.

[37] Herman A., Herman A. P., Domagalska B. W., Młynarczyk A. (2013). Essential oils and herbal extracts as antimicrobial agents in cosmetic emulsion. *Indian Journal of Microbiology*.

[38] Jentzsch P. V., Ramos L. A., Ciobotă V. (2015). Handheld Raman spectroscopy for the distinction of essential oils used in the cosmetics industry. *Cosmetics*.

[39] Kejlova K., Jirova D., Bendova H., Gajdoš P., Kolářová H. (2010). Phototoxicity of essential oils intended for cosmetic use. *Toxicology in Vitro*.

[40] Lee Y. (2023). Antifungal activity of Bulgarian rose damascena oil against vaginitis-causing opportunistic fungi.

